# The Association between Perceived Discrimination and Mental Health of Wage Workers with Disabilities: Findings from the Panel Survey of Employment for the Disabled 2016–2018

**DOI:** 10.3390/ijerph19148541

**Published:** 2022-07-13

**Authors:** Hyeon Ji Lee, Wonjeong Jeong, Doukyoung Chon, Jae-Hyun Kim, Jong Youn Moon

**Affiliations:** 1 Institute for Digital Life Convergence, Dankook University, Cheonan 31116, Korea; hyeonji@dankook.ac.kr (H.J.L.); cdytgtg@naver.com (D.C.); 2 Department of Preventive Medicine, Gachon University College of Medicine, Incheon 21565, Korea; wjjeong@gachon.ac.kr; 3 Artificial Intelligence and Big-Data Convergence Center, Gil Medical Center, Gachon University College of Medicine, Incheon 21565, Korea; 4 Department of Health Administration, Dankook University, Cheonan 31116, Korea

**Keywords:** disabled persons, social discrimination, workplace, psychological distress, depression

## Abstract

Despite efforts to integrate society, persons with disabilities (PWD) still experience considerable discrimination. Therefore, this study examined the association between experiences of discrimination and stress/depressive symptoms in wage working PWD. This study used data from the Panel Survey of Employment for the Disabled 2016–2018 in South Korea. This study included 1566 wage working PWD aged 15–64. The dependent variable was stress and depressive symptoms, and the independent variable was the experience of discrimination due to disability in daily life (Never, Rarely, Often, and Regularly) and the experience of discrimination at the workplace (0, 1, 2, ≥3). This study used a generalized estimating equations model to consider the repeated measurement data. Wage working PWD who experienced more discrimination in their daily life due to disability and at workplaces showed a higher odds ratio (OR) of stress and depressive symptoms than those who did not experience discrimination. As a result of the analysis including both discrimination experiences, those who always experienced discrimination due to disability in daily life had the highest OR to stress and depression (OR = 2.64, 95% Confidence Interval (CI): 1.37–5.08; OR = 4.96, 95% CI: 2.58–9.56, respectively). According to the experience at workplaces, wage working PWD who faced discrimination by two factors (OR = 1.66, 95% CI: 1.22–2.25) had the highest OR of stress, and those who experienced three or more factors had the highest OR of depressive symptoms (OR = 1.33, 95% CI: 0.83–2.11). Discrimination due to disability in daily life was more associated with the mental distress of working PWD than discrimination at workplaces. For the mental health of working PWD, not only policies or systems to eliminate discrimination in the workplace, but also overall social integration efforts based on improving awareness, are needed so that they do not experience discrimination in their daily life.

## 1. Introduction

There are over 1 billion persons with disabilities (PWD) worldwide, and the number is expected to increase in the future as the population ages, chronic disease prevalence rises, and exposure to risks, such as accidents, increases [1]. In the past, from the point of view of the charity model and the medical model, disability was considered as an individual’s physical defect that needed to be overcome, and PWD were viewed as being in need of help from others [2,3,4]. However, disability is an evolving concept, and a new paradigm for disability has emerged [5,6]. The definition of disability is complex and difficult to clarify, but based on bio-psycho-social models, disability is defined as the interaction between physical or mental impairment leading to activity limitation in an individual and the environment surrounding them in modern society [1,7]. Disability is not limited to an individual problem but can be defined and perceived differently through the surrounding environment.

The Convention on the Rights of Persons with Disabilities (CRPD) [5] and the enactment of laws to guarantee human rights, including the Disability Discrimination Act (DDA) [8,9], were intended to raise awareness of disabilities and the human right of the PWD and to strive for social integration. In South Korea, like in some other countries, there is a DDA [8] and mandatory employment quota system for the PWD, requiring a minimum percentage of employment for the PWD [10]. However, despite such efforts to integrate the society, discrimination experienced by the disabled remains. According to the results of the 2020 survey on the disabled in South Korea, 63.5% of the respondents said they had experienced discrimination. This decreased from 79.9% in 2017, but it is evident that a significant number of people with disabilities are still experiencing discrimination [11]. In addition, according to the results of the sample of the entire population in South Korea, the experience of discrimination in each situation is at least 2% and up to 11%, which is different from the results of the survey only for the PWD [12].

Discrimination is one of the major obstacles to achieving social integration and can be defined as the unfair or biased treatment of particular individuals or groups [13]. According to previous studies, discrimination can have a negative impact on living a healthy life, because it is psychologically and mentally harmful. Furthermore, most of the previous studies focused on the effects of race/ethnicity, sexual orientation, gender, and workplace discrimination on mental health rather than disability [14,15,16,17]. However, PWD are also experiencing discrimination [6,18], and there is a need for attention and management of their mental health, because they may be more vulnerable to clinical depression as well as other psychological problems compared to persons without disabilities (PW/OD) [19,20]. Notably, as the employment rate of the PWD rises, the working environment of the PWD is also increasingly drawing attention. Several previous studies have studied discrimination experienced by the PWD in the workplace and reported that the PWD experience more perceived discrimination than the PW/OD [21,22,23]. Even in nationally representative survey results in South Korea, the proportion of PWD who experienced discrimination at work was at least 12% to 19% by reason of discrimination [24], whereas only about 9% of the PW/OD experienced discrimination at work [12,25]. As the employment of PWD increases [26], it is necessary to study discrimination against working PWD and the resulting mental health for their health management.

Therefore, this study aims to examine the perceived discrimination by wage working PWD—discrimination experiences due to disability in daily life and discrimination experiences in the workplace—and to observe the association between the perceived discrimination and stress/depressive symptoms in wage working PWD. Ultimately, based on the hypothesis that experiences of discrimination are associated with poor mental health, by clarifying the association of discrimination in daily lives and workplace with stress and depressive symptoms, it aims to emphasize the importance of developing disability labor policies that can better deal with the mental health of the wage working PWD.

## 2. Materials and Methods

### 2.1. Study Sample

Data were collected from the second wave of the Panel Survey of Employment for the Disabled (PSED) between 2016 and 2018 (second wave 1st–3rd year). The second wave of the PSED was started in 2016 by selecting new panel survey targets conducted by Korea Employment Agency for the Disabled/Employment Development Institute (KEAD EDI). Among the registered PWD according to the Welfare of Persons with Disabilities Act, 4577 people were selected using two-phase sampling from the working-age range of 15–64 as of 15 May 2016, considering the region, age, disability type, disability grade, economic activity status, etc. The PSED is the nationally representative longitudinal survey of individuals with registered disabilities in South Korea, and nationwide data were collected using a computer-assisted personal interviewing program [27]. The PSED was designed to provide useful data for understanding the economic activities of PWD related to their employment [28]. In the baseline year (2016), only wage working PWD were extracted from all PWD to examine discrimination in their work and lives (n = 1763). Among them, participants were additionally excluded with missing information until 2018. Finally, 1566 wage working PWD were selected as study participants at baseline.

### 2.2. Independent Variables

The independent variables were the experiences of discrimination of wage working PWD. Experience of discrimination was considered in two different circumstances (i.e., due to disability in daily life or at the workplace). Experience of discrimination due to disability in daily life was measured by the participants’ response to “Experience of discrimination due to disability in daily lives”. Possible responses were “Never”, “Rarely”, “Often”, and “Regularly”. Experience of discrimination at the workplace was measured by the participants’ response to different reasons for experiencing discrimination at workplace (i.e., sex, age, disability, education level, region of origin, employment status, work expertise, rank). Participants gained a score of one for responding “Yes” to a reason of discrimination at the workplace, and a score of zero for responding “No”. The scores of the eight items were summed to generate four categories: “0”, “1”, “2”, “≥3”.

### 2.3. Dependent Variables

The dependent variables were stress and depressive symptoms of wage working PWD. Stress and depressive symptoms were measured by single questions in PSED. Stress was assessed by a question, “amount of stress in daily lives”, on a Likert scale. Possible responses were 1: “Not at all”, 2: “Not very much”, 3: “Insignificant”, 4: “Moderate”, and 5: “High”. Responses 1~3 were categorized as “No”, and 4~5 were categorized as “Yes” [29]. Depressive symptom was measured by a question, “Experience of feeling sad or hopeless enough to interfere with daily life for two weeks or more within the past year”, possible responses were “Yes” and “No”. Participants’ responses were used as a depressive symptom variable [30,31].

### 2.4. Control Variables

Covariates used in the study were age (15–29, 30–39, 40–49, 50–59, >59), residential region (metropolitan, urban, rural), marital status (married, single, divorced, or separated), self-rated health (poor, good), smoking status (current smoker, former smoker, non-smoker), alcohol consumption (drinker, former drinker, non-drinker), year (2016, 2017, 2018), depressive symptom, stress, disability grade, and disability type. Depressive symptom and stress were controlled for depending on the dependent variable (i.e., control for depressive symptom when stress was set as the dependent variable and vice versa). The disability type and disability grade used the responses to the survey according to the related law. According to the law, the types of disabilities are classified into a total of 15 types (disabilities of physical, brain lesion, facial, auditory, speech, kidney, heart, respiratory organs, liver, intellectual, developmental, and psychiatric, intestinal/urinary fistula, and epilepsy) [30,32] and using them, the study participants’ disability types were classified into physical disabilities and other groups. The disability grade is classified into a total of 6 grades in the law, and in this study, it was divided into 2 groups as follows: severe (levels 1 to 3) and moderate (levels 4 to 6) [31].

### 2.5. Analytical Approach and Statistics

The differences between the characteristics of the respondents were examined using the chi-square test. *p*-value < 0.05 was considered statistically significant. Participants who responded repeatedly three times were included in the study, and all variables (independent, dependent, and control variables) were measured three times. Therefore, a generalized estimating equation (GEE) model was used to examine the association between perceived discrimination in daily life and the workplace and mental health—stress and depression of the wage working PWD. The GEE model was used to analyze the variation within individuals of repeated measurement variables [29,33]. For the analysis using the GEE model, the SAS procedure “PROC GENMOD” was used, and the best model was selected by checking the working correlation structure [34,35]. Analyses with GEE were expressed as odds ratio (OR) and 95% confidence interval (CI). All statistical analyses were performed using SAS statistical software package version 9.4 (SAS Institute Inc., Cary, NC, USA).

## 3. Results

Table 1 shows the general characteristics of the study participants. Of the 1566 subjects, 56% responded that they had stress, and 10.7% reported depressive symptoms; 54.3% of those who experienced discrimination due to disability in their daily life and 85.6% of those who experienced discrimination in workplaces. For those who experienced discrimination due to disability in their daily life, the more frequently they experienced discrimination, the more likely they reported symptoms of stress and depression. In terms of the experience of discrimination at work, the more factors of perceived discrimination, the more they tended to answer that they had symptoms of stress and depression (Table 1).

Table 2 and Table 3 are the results, including covariates (Table 2 and Table 3). Table 2 shows the association between experience of discrimination due to disability in their daily life and stress/depressive symptoms. Table 3 shows the association between experiences of discrimination at the workplace and stress/depressive symptoms. Compared to those who did not experience discrimination due to disability in daily life, as more discrimination was experienced, the level of stress tended to increase. The “Regularly” group had the highest OR of stress and was statistically significant (OR = 3.16, 95% CI: 1.65–6.05). Depressive symptoms were also more likely to be experienced as the discriminatory experiences became more frequent, and were all statistically significant (OR = 1.66, 95% CI: 1.30–2.14; OR = 2.34, 95% CI: 1.66–3.31; OR = 6.02 95% CI: 3.18–11.41, respectively). Regarding the experience of discrimination in the workplace, the group with two discriminatory factors had the highest OR of stress (OR = 1.762, 95% CI: 1.306–2.376), followed by the three or more groups (OR = 1.756, 95% CI: 1.276–2.415). The higher the number of discriminatory factors, the higher the OR of depressive symptoms, and it was statistically significant only in the three or more group (OR = 1.87, 95% CI: 1.19–2.92).

Table 4 is the result of the analysis including both discrimination experiences due to disability in daily life and discrimination experiences at workplaces. Compared to the group with no experience of discrimination, the higher the discrimination intensity, the higher the OR of stress and depression. Those who always experienced discrimination due to disability in daily life, had the highest OR of stress and depression (OR = 2.64, 95% CI: 1.37–5.08; OR = 4.96, 95% CI: 2.58–9.56, respectively). According to the discrimination experience at workplaces, wage working PWD who experienced discrimination by two factors (OR = 1.66, 95% CI: 1.22–2.25) had the statistically significantly highest OR of stress. For depressive symptoms, the OR was highest among those who experienced three or more factors, but was not statistically significant (OR = 1.33, 95% CI: 0.83–2.11) (Table 4).

## 4. Discussion

This study investigated the discrimination experienced by wage working PWD due to their disability in their daily life and the discrimination they experienced at the workplace to examine the association between their experiences of discrimination and stress/depressive symptoms. The purpose of this study was to clarify the association of discrimination in daily lives and the workplace with stress and depressive symptoms to emphasize the importance of developing disability policies that could better address mental health for the PWD. Our findings show that those who perceived more discrimination tended to experience greater levels of stress and depressive symptoms. Especially compared to those who never experienced workplace discrimination, those who experienced workplace discrimination more than three times had higher odds of stress and depressive symptoms. These results could emphasize the importance of managing mental health, especially for working PWD.

In this study, more than half of the respondents reported that they had experienced discrimination due to disability in their daily life or discrimination in their workplace. Furthermore, in another survey of the PWD in South Korea, there were more PWD who answered having experienced discrimination than those who did not [11], showing consistent findings with the current study. These results show that, despite the efforts for social integration through the CRPD [5] or the DDA [8,9], the PWD still experience and are aware of discrimination, and the number is significant. Particularly, in this study, 85.6% of respondents reported experiencing discrimination in the workplace. In addition, some of them experienced discrimination due to multiple factors. These results show that the discrimination experienced by the wage working PWD in the workplace can include not only discrimination due to disability, but also discrimination based on other reasons.

Previous studies have shown that the PWD are more susceptible to discrimination than PW/OD [21,22,23] and have been associated with unhealthy conditions, including mental health [19,20]. In addition, the ratio of experiencing discrimination in the workplace was higher in PWD than in PW/OD [12,24,25]. Therefore, this study examined the association between discrimination experience and mental health in the wage working PWD. As a result of analyzing the association between the discrimination experienced due to disability in daily life and the symptoms of stress/depression, it was found that the more frequently discrimination was recognized, the more stress/depression symptoms were experienced. In particular, wage working PWD who perceived discrimination regularly were fairly more likely to feel psychological distress.

Furthermore, discrimination based on various factors, regardless of disability, adversely affects overall health [36,37,38], including mental health [14,15,16,17]. Therefore, this study examined the association between various discriminations experienced at workplaces and mental health. According to the current findings, as the PWD perceived discrimination due to more factors at workplaces, more stress/depressive symptoms were reported. These results are similar to those of previous studies of the PWD [39] and PW/OD [17], indicating the beneficial effect of eliminating discrimination due to any cause for health and well-being.

Moreover, the experience of discrimination due to disability in daily life and the experience of discrimination at the workplace were simultaneously put into the analysis to examine the association between the experience of discrimination and mental health in this study. According to the analysis of each discrimination experience, perceiving discrimination was more negatively associated with mental health. Particularly, it seemed that the experience of discrimination due to disability in daily life was more related to the mental distress of the wage working PWD than discrimination in the workplace. Additionally, discrimination experiences due to disability in daily life were more likely to affect depressive symptoms than stress, and discrimination experiences at workplaces tended to affect stress more than depressive symptoms. A previous study has reported a mechanism by which social defeat-induced persistent stress causes transitions to chronic depression [40]. Accordingly, the possible interpretation is that in daily life, discrimination experiences occur repeatedly and persist for a long time, so they are more likely to affect not only stress, but also depressive symptoms. Moreover, discrimination experiences at work tend to be associated with a higher level of stress, and this could also lead to depression. Therefore, it is a necessity to prevent stress due to discrimination and to manage depression.

This study shows that the working PWD still experience discrimination and supports the results of previous studies that the perceived discrimination of the working PWD have a negative effect on their mental health. Therefore, it suggests that discrimination of all causes that the working PWD perceive and experience needs to be eliminated as much as possible to ensure the optimal mental health and, ultimately, the overall health of the disabled. Additionally, by reporting that discrimination experiences due to disability in daily life are related to the worse mental health of the working PWD rather than discrimination experiences in the workplace, the previous studies on perceptions and mental health of people with disabilities are expanded. 

This study has some limitations. First, as the survey was conducted only on registered PWD, unregistered PWD were not considered. In addition, since only the working PWD were extracted and studied to examine the experience of discrimination in daily life and workplaces, there is a limit to expanding the results of this study to the entire PWD. Second, due to the limitation of the survey questionnaire used in the study, the variable using structured tools in the study could not be reflected, and there may be a response bias of the study participants. In particular, the discrimination experience distinguished between experiences due to disability in daily life and experiences at workplaces, but the questionnaire on these two variables was not unified, so the operational definition for the study was made in different ways. Therefore, in future research, it is necessary to conduct research by precisely quantifying the experience of discrimination using standardized indicators. Furthermore, dependent variables (stress/depressive symptoms) used in this study were evaluated using one questionnaire item. However, previous studies have validated the possible benefits of using subjective measures [30,41,42], abating this limitation. Lastly, the research results mean only associations, not causal relationships.

## 5. Conclusions

This study shows the association between the experience of discrimination of working PWD and their mental distress. For the mental health of working PWD, not only policies or systems to eliminate discrimination in the workplace, but also overall social integration efforts based on improving the awareness of members of the society, including systems, are needed so that they do not experience discrimination in their daily lives.

## Figures and Tables

**Table 1:** General characteristics of study participants included for analysis at baseline.

Variables	Total		Stress					Depressive Symptom				
			No		Yes		*p*-Value	No		Yes		*p*-Value
	N	%	N	%	N	%		N	%	N	%	
**Experience of discrimination due to disability in daily life**							<0.0001					<0.0001
Never	716	45.7	353	49.3	363	50.7		665	92.9	51	7.1	
Rarely	600	38.3	253	42.2	347	57.8		525	87.5	75	12.5	
Often	220	14.1	77	35.0	143	65.0		188	85.5	32	14.6	
Regularly	30	1.9	6	20.0	24	80.0		21	70.0	9	30.0	
**Experience of discrimination at workplace**							<0.0001					<0.0001
0	226	14.4	107	47.4	119	52.7		203	89.8	23	10.2	
1	1124	71.8	521	46.4	603	53.7		1031	91.7	93	8.3	
2	119	7.6	32	26.9	87	73.1		94	79.0	25	21.0	
≥3	97	6.2	29	29.9	68	70.1		71	73.2	26	26.8	
**Gender**							0.4108					0.3366
Male	1182	75.5	527	44.6	655	55.4		1061	89.8	121	10.2	
Female	384	24.5	162	42.2	222	57.8		338	88.0	46	12.0	
**Age**							0.1393					0.6582
15–29	231	14.8	116	50.2	115	49.8		205	88.7	26	11.3	
30–39	483	30.8	211	43.7	272	56.3		440	91.1	43	8.9	
40–49	515	32.9	209	40.6	306	59.4		456	32.6	59	11.5	
50–59	233	14.9	109	46.8	124	53.2		207	88.8	26	11.2	
>59	104	6.6	44	42.3	60	57.7		91	87.5	13	12.5	
**Residential region**							0.3095					0.0004
Metropolitan	327	20.9	145	44.3	182	55.7		283	86.5	44	13.5	
Urban	411	26.3	193	47.0	218	53.0		388	94.4	23	5.6	
Rural	828	52.9	351	42.4	477	57.6		728	87.9	100	12.1	
**Marital status**							0.1184					<0.0001
Married	862	55.0	362	42.0	500	58.0		789	91.5	73	8.5	
Single	525	33.5	250	47.6	275	52.4		467	89.0	58	11.1	
Divorce, separated	179	11.4	77	43.0	102	57.0		143	79.9	36	20.1	
**Self-rated health**							<0.0001					<0.0001
Poor	443	28.3	139	31.4	304	68.6		367	82.8	76	17.2	
Good	1123	71.7	550	49.0	573	51.0		1032	91.3	81	8.1	
**Smoking status**							0.0958					0.0394
Current smoker	451	28.8	180	39.9	271	60.1		390	86.5	61	13.5	
Former smoker	359	22.9	159	44.3	200	55.7		320	89.1	39	10.9	
Non-smoker	756	48.3	350	46.3	406	53.7		689	91.1	67	8.9	
**Alcohol consumption**							0.2759					0.4848
Drinker	905	57.8	384	24.5	521	33.3		807	89.2	98	10.8	
Former drinker	253	16.2	112	44.3	141	55.7		222	87.8	31	12.3	
Non-drinker	407	26.0	192	47.2	215	52.8		369	90.7	38	9.3	
**Depressive symptom/Stress**							<0.0001					<0.0001
Yes/No	167	10.7	28	16.8	139	83.2		661	95.9	28	4.1	
No/Yes	1399	89.3	661	47.3	738	52.8		738	84.2	139	15.9	
**Disability grade**							0.6209					0.1956
Severe	404	25.8	182	45.1	222	55.0		354	87.6	50	12.4	
Moderate	1162	74.2	507	43.6	655	56.4		1045	89.9	117	10.1	
**Disability type**							0.8740					0.0450
Physical disability	892	57.0	394	44.2	498	55.8		809	90.7	83	9.3	
Other	674	43.0	295	43.8	379	56.2		590	87.5	84	12.5	
**Total**	**1566**	**100**	**689**	**44.0**	**877**	**56.0**		**1399**	**89.3**	**167**	**10.7**	

**Table 2:** Association between experience of discrimination due to disability in their daily life and stress/depressive symptoms.

Variables	Stress			Depressive Symptom		
	OR	95% CI	*p*-Value	OR	95% CI	*p*-Value
**Experience of discrimination due to disability in daily life**						
Never	1.00	–		1.00	–	
Rarely	1.04	(0.91–1.18)	0.5732	1.66	(1.30–2.14)	<0.0001
Often	1.46	(1.17–1.83)	0.0007	2.34	(1.66–3.31)	<0.0001
Regularly	3.16	(1.65–6.05)	0.0005	6.02	(3.18–11.41)	<0.0001
**Gender**						
Male	0.94	(0.80–1.12)	0.5026	0.54	(0.40–0.74)	0.0001
Female	1.00	–		1.00	–	
**Age**						
15–29	1.64	(1.20–2.25)	0.0021	1.33	(0.72–2.45)	0.3570
30–39	1.58	(1.22–2.05)	0.0005	1.40	(0.84–2.34)	0.1995
40–49	1.59	(1.24–2.04)	0.0003	1.47	(0.90–2.39)	0.1233
50–59	1.27	(0.97–1.64)	0.0771	1.54	(0.94–2.54)	0.0892
> 59	1.00	–		1.00	–	
**Residential region**						
Metropolitan	0.99	(0.85–1.16)	0.8979	0.77	(0.58–1.03)	0.0760
Urban	1.01	(0.87–1.17)	0.9007	0.44	(0.32–0.60)	<0.0001
Rural	1.00	–		1.00	–	
**Marital status**						
Married	1.27	(1.03–1.55)	0.0225	0.44	(0.33–0.60)	<0.0001
Single	0.91	(0.72–1.16)	0.4355	0.74	(0.51–1.07)	0.1129
Divorce, separated	1.00	–		1.00	–	
**Self-rated health**						
Poor	2.03	(1.75–2.35)	<0.0001	2.47	(1.96–3.12)	<0.0001
Good	1.00	–		1.00	–	
**Smoking status**						
Current smoker	1.19	(1.00–1.42)	0.0491	1.84	(1.32–2.57)	0.0003
Former smoker	0.99	(0.83–1.18)	0.8913	1.34	(0.94–1.92)	0.1026
Non-smoker	1.00	–		1.00	–	
**Alcohol consumption**						
Drinker	1.16	(0.98–1.37)	0.0861	1.29	(0.94–1.78)	0.1178
Former drinker	1.06	(0.86–1.29)	0.5943	1.53	(1.06–2.21)	0.0231
Non-drinker	1.00	–		1.00	–	
**Depressive symptom/Stress**						
Yes/No	2.91	(2.24–3.78)	<0.0001	0.35	(0.27–0.45)	<0.0001
No/Yes	1.00	–		1.00	–	
**Disability grade**						
Severe	0.87	(0.75–1.02)	0.0832	0.91	(0.69–1.19)	0.4879
Moderate	1.00	–		1.00	–	
**Disability type**						
Physical disability	1.01	(0.88–1.15)	0.9204	0.73	(0.58–0.93)	0.0108
Other	1.00	–		1.00	–	
**Year**						
2016	0.98	(0.85–1.14)	0.8321	1.46	(1.11–1.91)	0.0059
2017	1.03	(0.89–1.19)	0.6663	1.11	(0.83–1.47)	0.4810
2018	1.00	–		1.00	–	

**Table 3:** Association between experiences of discrimination at the workplace and stress/depressive symptoms.

Variables	Stress			Depressive Symptom		
	OR	95% CI	*p*-Value	OR	95% CI	*p*-Value
**Experience of discrimination at workplace**						
0	1.00	–		1.00	–	
1	1.03	(0.87–1.23)	0.7113	0.75	(0.54–1.04)	0.0843
2	1.76	(1.31–2.38)	0.0002	1.55	(1.00–2.42)	0.0506
≥3	1.76	(1.28–2.42)	0.0005	1.87	(1.19–2.92)	0.0064
**Gender**						
Male	0.95	(0.80–1.13)	0.5690	0.58	(0.42–0.78)	0.0004
Female	1.00	–		1.00	–	
**Age**						
15–29	1.71	(1.24–2.34)	0.0010	1.43	(0.78–2.62)	0.2503
30–39	1.61	(1.24–2.09)	0.0003	1.48	(0.89–2.48)	0.1328
40–49	1.62	(1.26–2.07)	0.0002	1.53	(0.94–2.49)	0.0857
50–59	1.28	(0.98–1.66)	0.0660	1.60	(0.97–2.63)	0.0661
> 59	1.00	–		1.00	–	
**Residential region**						
Metropolitan	1.01	(0.86–1.18)	0.9293	0.80	(0.60–1.05)	0.1091
Urban	1.01	(0.88–1.17)	0.8542	0.44	(0.33–0.60)	<0.0001
Rural	1.00	–		1.00	–	
**Marital status**						
Married	1.28	(1.04–1.56)	0.0175	0.42	(0.31–0.58)	<0.0001
Single	0.91	(0.71–1.15)	0.4234	0.73	(0.50–1.06)	0.0957
Divorce, separated	1.00	–		1.00	–	
**Self-rated health**						
Poor	2.04	(1.76–2.37)	<0.0001	2.60	(2.06–3.27)	<0.0001
Good	1.00	–		1.00	–	
**Smoking status**						
Current smoker	1.17	(0.98–1.39)	0.0812	1.69	(1.22–2.35)	0.0018
Former smoker	0.98	(0.82–1.17)	0.8395	1.33	(0.93–1.89)	0.1148
Non-smoker	1.00	–		1.00	–	
**Alcohol consumption**						
Drinker	1.13	(0.95–1.34)	0.1563	1.22	(0.89–1.67)	0.2226
Former drinker	1.03	(0.84–1.25)	0.8047	1.41	(0.98–2.02)	0.0657
Non-drinker	1.00	–		1.00	–	
**Depressive symptom/Stress**						
Yes/No	2.90	(2.23–3.76)	<0.0001	0.35	(0.27–0.45)	<0.0001
No/Yes	1.00	–		1.00	–	
**Disability grade**						
Severe	0.91	(0.78–1.06)	0.2069	1.10	(0.84–1.44)	0.4826
Moderate	1.00	–		1.00	–	
**Disability type**						
Physical disability	1.00	(0.88–1.15)	0.9486	0.73	(0.58–0.93)	0.0119
Other	1.00	–		1.00	–	
**Year**						
2016	1.00	(0.86–1.16)	0.9876	1.51	(1.16–1.98)	0.0023
2017	1.03	(0.89–1.19)	0.7083	1.11	(0.83–1.47)	0.4778
2018	1.00	–		1.00	–	

**Table 4:** Results of the analysis including both discrimination experiences and stress/depressive symptoms.

Variables	Stress			Depressive Symptom		
	OR	95% CI	*p*-Value	OR	95% CI	*p*-Value
**Experience of discrimination due to disability in daily life**						
Never	1.00	–		1.00	–	
Rarely	0.98	(0.86–1.12)	0.7827	1.59	(1.22–2.05)	0.0005
Often	1.31	(1.04–1.64)	0.0206	2.10	(1.46–3.01)	<0.0001
Regularly	2.64	(1.37–5.08)	0.0036	4.96	(2.58–9.56)	<0.0001
**Experience of discrimination at workplace**						
0	1.00	–		1.00	–	
1	1.02	(0.86–1.22)	0.7859	0.67	(0.48–0.93)	0.0182
2	1.66	(1.22–2.25)	0.0012	1.15	(0.72–1.82)	0.5540
≥3	1.62	(1.17–2.24)	0.0040	1.33	(0.83–2.11)	0.2379
**Gender**						
Male	0.95	(0.80–1.12)	0.5234	0.55	(0.40–0.75)	0.0001
Female	1.00	–		1.00	–	
**Age**						
15–29	1.69	(1.23–2.32)	0.0012	1.38	(0.75–2.55)	0.2993
30–39	1.60	(1.23–2.07)	0.0004	1.47	(0.88–2.46)	0.1450
40–49	1.61	(1.25–2.06)	0.0002	1.50	(0.92–2.45)	0.1013
50–59	1.28	(0.98–1.66)	0.0689	1.59	(0.96–2.62)	0.0707
>59	1.00	–		1.00	–	
**Residential region**						
Metropolitan	1.00	(0.85–1.17)	0.9958	0.78	(0.59–1.04)	0.0874
Urban	1.01	(0.88–1.17)	0.8747	0.44	(0.32–0.59)	<0.0001
Rural	1.00	–		1.00	–	
**Marital status**						
Married	1.28	(1.05–1.57)	0.0161	0.44	(0.33–0.60)	<0.0001
Single	0.91	(0.71–1.15)	0.4245	0.74	(0.51–1.08)	0.1155
Divorce, separated	1.00	–		1.00	–	
**Self-rated health**						
Poor	2.03	(1.75–2.35)	<0.0001	2.47	(1.96–3.12)	<0.0001
Good	1.00	–		1.00	–	
**Smoking status**						
Current smoker	1.18	(0.99–1.41)	0.0600	1.78	(1.28–2.49)	0.0007
Former smoker	0.99	(0.83–1.18)	0.9082	1.36	(0.95–1.94)	0.0902
Non-smoker	1.00	–		1.00	–	
**Alcohol consumption**						
Drinker	1.15	(0.97–1.36)	0.1119	1.29	(0.93–1.78)	0.1235
Former drinker	1.05	(0.86–1.28)	0.6483	1.50	(1.04–2.18)	0.0302
Non-drinker	1.00	–		1.00	–	
**Depressive symptom/Stress**						
Yes/No	2.81	(2.16–3.65)	<0.0001	0.36	(0.28–0.47)	<0.0001
No/Yes	1.00	–		1.00	–	
**Disability grade**						
Severe	0.87	(0.74–1.01)	0.0727	0.93	(0.70–1.23)	0.6058
Moderate	1.00	–		1.00	–	
**Disability type**						
Physical disability	1.01	(0.88–1.15)	0.8878	0.74	(0.58–0.95)	0.0163
Other	1.00	–		1.00	–	
**Year**						
2016	0.98	(0.85–1.14)	0.7869	1.44	(1.10–1.89)	0.0076
2017	1.03	(0.89–1.19)	0.6734	1.11	(0.83–1.47)	0.4743
2018	1.00	–		1.00	–	

## Data Availability

The dataset is available on the PSED website: https://edi.kead.or.kr/ENG_Index.do (accessed on 30 June 2022).
